# Depression in Diabetic Patients: What Is the Link With Eating Disorders? Results of a Study in a Representative Sample of Patients With Type 1 Diabetes

**DOI:** 10.3389/fpsyt.2022.848031

**Published:** 2022-06-16

**Authors:** Federica Pinna, Federico Suprani, Valeria Deiana, Lorena Lai, Mirko Manchia, Pasquale Paribello, Giulia Somaini, Enrica Diana, Eraldo Francesco Nicotra, Fernando Farci, Mariangela Ghiani, Rossella Cau, Marta Tuveri, Efisio Cossu, Elena Loy, Andrea Crapanzano, Paola Grassi, Andrea Loviselli, Fernanda Velluzzi, Bernardo Carpiniello

**Affiliations:** ^1^Section of Psychiatry, Department of Medical Sciences and Public Health, University of Cagliari, Cagliari, Italy; ^2^Unit of Clinical Psychiatry, University Hospital Agency of Cagliari, Cagliari, Italy; ^3^Department of Pharmacology, Dalhousie University, Halifax, NS, Canada; ^4^Department of Education, Psychology and Philosophy, University of Cagliari, Cagliari, Italy; ^5^Unit of Diabetology, Azienda Sanitaria Locale Cagliari, Quartu Sant’Elena, Italy; ^6^Endocrinology and Diabetes Unit, University Hospital Agency of Cagliari, Cagliari, Italy; ^7^Department of Counseling, San Francisco State University, San Francisco, CA, United States; ^8^Endocrinology and Obesity Unit, Department of Medical Sciences and Public Health, University of Cagliari, Cagliari, Italy

**Keywords:** depression, diabetes, eating disorders, Disturbed Eating Behaviors, comorbidity

## Abstract

**Background and Purpose:**

Comorbidity between diabetes and depression, and diabetes and eating disorders (ED) conveys significant diagnostic, clinical and therapeutic implications. The present study was conducted on a sample of adult outpatients affected by Type 1 Diabetes (T1DM) to assess lifetime prevalence of ED; current prevalence of depression and Disturbed Eating Behaviors (DEB) and their impact on glycemic control. We hypothesized that patients with depression would have higher rates of lifetime ED and current DEB. We hypothesized a significant and independent association between DEB and the prevalence of depression.

**Materials and Methods:**

The study was carried out using a cross-sectional design in a sample of 172 diabetic patients with T1DM aged from 17 to 55 years. Lifetime prevalence of ED according to DSM-5 criteria was assessed by means of the Module H modified of the Structured Clinical Interview for DSM-IV Axis I Disorder (SCID-I). The following questionnaires were used: Beck Depression Inventory–IA version (BDI-IA) and Diabetes Eating Problems Survey—Revised (DEPS-R), to assess respectively the current presence of depression and DEB. Socio-demographic, clinical, and laboratory data were also collected.

**Results:**

High rates of depression (35.5%) and DEB (19.2%) were observed in our sample of 172 adult outpatients with T1DM. Lifetime history of ED was present in 20.9% of the sample and was more frequently diagnosed in patients with current depression (34.4% vs. 13.9%, *p* = 0.002). Higher levels of DEB at DEPS-R significantly increased the odds of depression (adjOR: 1.09; 95% CI: 1.03–1.15; *p* = 0.003). The presence of DEB was associated with poor glycemic control. On the other hand, no association was found between depression and metabolic compensation.

**Conclusion:**

Adult patients with T1DM and depression should be screened for ED and DEB. Treating DEB could positively impact both mood and glycemic control in this population. Further studies should be carried out on a larger patient population using a longitudinal design and an accurate method of evaluation to explore the complex relationship between diabetes, depression, ED, and DEB. Future research should investigate treatment strategies for DEB in T1DM patients and their impact on both psychopathological and metabolic outcomes.

## Introduction

Depression, still today one of the most common psychiatric disorders encountered worldwide, produces a substantial burden on public health, representing one of the major causes of disability and generating a significant impact in terms of both healthcare costs and suffering of those affected (1). Estimated lifetime prevalence rates reported for the disease are approximately 15% in high-income countries, including Italy, and 11% in low to middle-income countries, with mean age at onset ranging from 24 to 26 years, and a 2-fold higher risk in females (1). The high burden produced by depression is correlated in part with an increased risk amongst depressed subjects of onset, persistence, and increased severity of a wide range of chronic conditions, including obesity, resulting in a further rise in levels of disability, premature death, and deterioration of quality of life (2–7).

The interaction between depression and medical conditions is highly complex and bidirectional, with each illness producing an impact on onset, course, prognosis, and treatment of the other (8). If, on the one hand, depression is approximately 2 to 3-fold more common in patients affected by a chronic physical condition (i.e., cancer, diabetes, heart conditions, and stroke), being manifested in approximately 20% of this patient population, at the same time, depressed subjects present with an increased incidence of chronic medical conditions compared to the general population (9, 10). In all cases, a condition of comorbidity between a clinically relevant depressive disorder and a somatic disorder is linked to a worse outcome of both the depressive and the somatic disorder (9). The latter finding underlines the importance of systematic assessment and recognition of levels of depression in people affected by somatic disorders, together with a correct management and appropriate treatment of any associated depression disorders.

Amongst the chronic physical conditions associated with depression, diabetes is of particular interest and relevance in view of evidence pointing to an exponential increase in cases of diabetes mellitus worldwide, which has been likened to a global epidemic of diabetes: a prevalence rate in the global population of 9.3% (463 million people) was estimated in 2019, which is likely to reach 10.2% (578 million people) by 2030 and 10.9% (700 million people) by 2045 (11). Based on the growing burden of this condition, even in developing countries, the World Health Organization has included diabetes amongst the chronic disorders requiring major investment in terms of prevention. Type 1 Diabetes Mellitus (T1DM), although manifested less frequently than Type 2 Diabetes Mellitus (T2DM) and representing 5–10% of the total burden of diabetes mellitus, is the most predominant endocrine disorder in pediatric and adolescent populations, and one of the most frequent chronic childhood disorders. From an epidemiological point of view, Sardinia, the second largest Italian island in the Mediterranean, has the second highest incidence of T1DM in the world after Finland (12).

Although both depression and anxiety are commonly observed disorders amongst patients affected by diabetes mellitus, prevalence data obtained for these conditions in the specific population, in particular T1DM, are scarce and somewhat lacking from a methodological viewpoint, and the direction of the association between diabetes and the psychopathological domain remains unclear due to the transversal design of the majority of studies conducted to date (13–15), while there are few studies with a longitudinal design (16, 17). Prevalence rates for depression were more than 3-fold higher in people affected by T1DM (12%, range 5.8–43.3% vs. 3.2%, range 2.7–11.4%) and up to 2-fold higher in those with T2DM (19.1%, range 6.5–33% vs. 10.7%, range 3.8–19.4%) vs. healthy controls; in all cases (T1DM, T2DM, and healthy controls), higher rates of depression were observed in women compared to men (14). Moreover, other studies focusing on the comorbidity between depression and T1DM reported an inconsistency in prevalence rates, although confirming the close relationship between depression and poor glycemic control and lack of self-care in diabetic patients (18, 19). Fisher et al. suggested that the cause of these discrepancies may lie in a methodological issue associated with a difficulty to distinguish between a depressive state and emotional distress linked to management of the chronic disease (20). The link between diabetes and depression has been examined in literature regarding both the concomitant psychological dimensions and potential reciprocal influencing effects of the two disorders. Indeed, depression manifested in subjects affected by diabetes tends to adhere to a recurrent or chronic course (18). The concomitant presence of mood disorders is correlated with a worse prognosis for diabetes (21), a less healthy lifestyle and a greater lack of diabetic self-care (22, 23), lower treatment compliance (24, 25), higher HbA_1_c (Glycated Hemoglobin A1c) (19, 24, 26–29), additional complications of diabetes (30, 31), a worsening of quality of life (24, 32) and higher mortality rates (31–33). In addition, the continual self-care required in the treatment of diabetes (blood sugar measurements, multiple doses of insulin, diet, exercise, etc.), is much harder to ensure in patients affected by concomitant depression.

It has been hypothesized that the etiopathogenesis of diabetes and depression may share several relevant aspects, i.e., stress and inflammation, with an increased propensity for T2DM (34), and that the presence of one of the two conditions may increase the risk of onset of the other (13, 35, 36). Moreover, other factors indicated as potentially capable of increasing the risk of depression in diabetic patients include a personal or family history of depression, exposure to stressful or traumatic events such as domestic violence, stress, and the burden, particularly in the case of T1DM, of being exposed to an early onset chronic disease, the presence of physical illnesses and other clinical factors (35, 36). Other authors suggest a series of physiopathological mechanisms common to both conditions, inducing us to consider the existence of a biological link between the two conditions: ranging from the effects produced by the circulating cytokines associated with autoimmune diabetes to the effects of a lack of insulin on neurogenesis and the neurotransmitter metabolism, to chronic hyperglycemia, iatrogenic hyperglycemia, and basal hyperactivity of the hypothalamic–pituitary–adrenal axis (37).

An additional confounding factor has been detected in the relationship between diabetes and psychiatric morbidity determined by the concomitant presence of depression and Eating Disorders (ED) (38, 39). To control diabetes, the patient is required to place particular emphasis on his or her nutrition, constantly monitoring the intake of food which correlates with the insulin dose prescribed. Overall, this persistent focus on food, fundamental in the management and regulation of diabetes, predisposes diabetic patients to developing ED, which are, in turn, more difficult to diagnose in these patients compared to the general population. There is evidence of ED, Disturbed Eating Behaviors (DEB), binge eating and symptoms of bulimia in both T1DM (40–47) and T2DM patients (47, 48), which generally exceed those observed in the general population (38, 40, 43, 45, 49). It has been demonstrated how both ED and DEB are capable of seriously impinging on physical health, particularly in diabetic subjects, and that the long-term complications of diabetes may be exacerbated by DEB and by an improper use of insulin which, by compromising metabolic regulation, result in increased mortality rates (38). Studies have shown an association between poor glycemic control and both ED (50) and subclinical disordered eating (51) in diabetic patients.

Longitudinal data on non-diabetic populations support a bidirectional relationship between depression and eating pathology where both conditions represent a risk factor for the other (52). Comorbidity is very common: more than 40% of individuals with ED reported comorbid mood disorders according to a recent narrative review on European studies (53). Identifying ED in subjects with depressive disorders is critical given the high rate of antidepressants resistance in this population (54), yet most people with ED go undetected even among psychiatric outpatients as they often seek care for comorbid mental disorders (55). Other authors have advocated the need to screen for disordered eating in subjects with Major Depressive Disorder (56).

To summarize there is evidence that: (i) the prevalence of both depression and DEB is higher in patients with diabetes compared to healthy controls (14, 40); (ii) both conditions have been associated with poor metabolic compensation (24, 51); (iii) there is a substantial and clinically relevant relationship between ED and depression in non-diabetic subjects, given the high rates of comorbidity (53), the difficulty to detect ED in patients with depression (56) and its impact on antidepressants response (54). Despite the aforementioned existing evidences, very few studies to date have examined the association between the depressive dimension and DEB in T1DM adult patients (40). Most of the literature has focused on adolescents and women (57), despite the fact the over 60% of depressive disorders has the onset after the age of 25 (58). Moreover, even if ED are more frequent in women (59), men presentations tend to be of similar severity (60), with a recent study finding males with ED more likely to have comorbid depression compared to females (61). Interestingly, a recent longitudinal study on adolescents and young adults with T1DM was conducted with the objective to investigate the directionality of effect linking DEB to depressive symptoms (62). To our knowledge, this was the only study assessing the directionality of disordered eating and depression in T1DM patients. Using cross-lagged analysis, the authors found that DEB predicted increases in depressive symptoms over time, while depressive symptoms at baseline did not predict DEB at follow-up (62). Other published literature mostly explored the association between DEB and depression in T1DM patients using cross-sectional design and/or bivariate analysis, without taking into account potential covariates and confounders (40, 57).

In the present study conducted on a sample of adult outpatients, males and females, affected by T1DM, we aimed to: (i) investigate the current prevalence of DEB and depression and the lifetime prevalence of ED; (ii) explore the relationship between DEB and depression; (iii) test the association of both depression and DEB with metabolic compensation. We hypothesized that:

1. the prevalence of current DEB and lifetime ED would be significantly higher in patients with depression compared to those without depression;
2. patients with depression would have significantly worse treatment adherence, higher HbA_1_c levels, longer duration of diabetes and more diabetes complications compared to those without;
3. DEB would be significantly positively correlated with HbA_1_c levels and BMI; DEB would be significantly higher in females compared to males, in patients with poor treatment adherence compared to patients with good treatment adherence, in those with diabetes complications compared to those without complications, and in subjects with a multiple daily injection insulin regimen (MDI) compared to those with continuous subcutaneous insulin therapy (CSIT).
4. the presence of DEB would significantly increase the odds of depression in the study population independently of metabolic covariates (HbA_1_c, continuous subcutaneous insulin therapy, treatment adherence, duration of diabetes, diabetes complications) and potential confounders (gender, age, education, employment, thyroid disorder, BMI, and physical activity).

To our knowledge, this is the first study to examine, in a sample comprehensive of adolescents and adults, males and females, the independent association between depression and DEB in T1DM by means of a detailed psychodiagnostic assessment tailored to the diabetic population.

## Materials and Methods

This was a cross-sectional study in a sample of insulin-treated diabetic outpatients with T1DM aged from 17 to 55 years old. An unselected sample of consecutive patients attending two specialist centers for the diagnosis and treatment of diabetes over a 4-month period (from June to September 2017) was assessed. These centers routinely provide a global medical, psychological, and psychopathological evaluation with a multidisciplinary approach. When necessary, psychological support or psychiatric assessment and intervention are made available. During the 4-month period of the study, the total number of patients registered to the participant centers, including all ages, types of diabetes mellitus (1/2) and hypoglycemic treatment (oral/insulin), was about 2000 (10% with T1DM). The primary study population was made up of 211 insulin-treated diabetic patients aged 17–55 years, 192 patients with a type 1, 19 patients with a type 2 diabetes, 108 males and 103 females. For the purposes of this study, only patients with T1DM were included in the final sample. Patients with a type 2 diabetes (*n* = 19) and who did not complete the psychometric assessment (*n* = 20) were excluded. The final sample included 172 participants, 86 males and 86 females. The data used for the purposes of this study derived from the routine assessment of psychopathology in the diabetic patients attending the specialist centers by means of questionnaires, specifically created to assess depression, anxiety, and DEB, and a structured clinical interview used to assess ED. After signing an informed consent form, socio demographic data, medical history and clinical data were collected by means of a specific data form. Lifetime prevalence of ED according to DSM-5 criteria was assessed by means of the Module H modified, according to DSM-5 criteria, of the Structured Clinical Interview for DSM-IV Axis I Disorder (SCID-I, Research Version, Non-Patient Edition) (63, 64). Structured Clinical Interviews (Module H modified) were conducted by residents in psychiatry trained in the use of the instrument by a senior psychiatrist (FP). The inter-rater reliability for module H, assessed using Cohen’s K before starting the study, remained substantial (K > 0.80) among the 8 clinicians who performed the assessments. The rationale for performing diagnosis with DSM-5 criteria was related to the increased ability of DSM-5 criteria to identify the presence of an ED and to formulate a “specified ED diagnosis” (65). The following questionnaires were used for the purposes of this study: Beck Depression Inventory–IA version (BDI-IA) (66) and Diabetes Eating Problems Survey—Revised (DEPS-R) (67).

### Instruments

#### Sociodemographic and Clinical Data Form

Collected data included age, gender (male or female), education (none, primary education, lower secondary education, upper secondary education, graduation), marital status (single, married, cohabiting, separated/divorced, widowed), age at onset and diabetes duration (years), weight, height, Body Max Index (BMI), value of hemoglobin A1c (HbA_1_c) (%), physical activity in the last 3 months (yes or no), continuous subcutaneous insulin therapy (CSIT) (yes or no), diabetes complications (yes or no), chronic pharmacological treatment (drugs and dosage), list of physical and psychiatric comorbidity, compliance to pharmacological and nutritional treatment according to the physician assessment (bad, poor, sufficient, good, excellent).

#### Beck Depression Inventory–IA Version

The BDI-IA questionnaire is a revised version of the original inventory developed by Beck in the 1970s and published in 1978. It is a self-administered scale comprising 21 questions or elements, each of which with four possible answers, aimed at exploring the events and symptoms experienced over the 2 weeks prior to assessment. Each answer is assigned a score ranging from zero to three based on the severity of the dimension assessed. Originally created to evaluate depression in patients attending mental health clinics, it was subsequently readapted for use in the context of a primary care setting. The ease of administration of the tool, taking from 5 to 10 min, underline its ease of insertion as part of a psychological or medical examination. The sum of scores obtained at each single item is indicative of the severity of depression, with high scores indicating increased severity of the depressive symptomatology. A score range of 0–9 indicates absence of or minimal depression; 10–18 indicates mild depression; 19–29 indicates moderate depression, and 30–63 indicates severe depression. The internal consistency for the BDI-IA was good, with a Cronbach’s alpha coefficient of around 0.85, indicating that the items on the inventory are highly correlated with each other (68).

#### Diabetes Eating Problems Survey—Revised

Diabetes eating problems survey—revised is a diabetes-specific measure of DEB. The scale is self-administered and comprises 16 items on a 6-point Likert, ranging from 0 to 5, based on frequency of the behavior (0 = never; 1 = rarely; 2 = sometimes; 3 = often; 4 = usually; 5 = always). It can be completed in less than 10 min. Higher scores indicate more DEB. The original instrument consisted of 28 items, but it was recently revised and shortened to the DEPS-R by Markowitz et al. (69). The Italian version of the tool, used for the purposes of this study, was found to have a good degree of reliability, a good homogeneity, and a good reproducibility in a sample of insulin-treated male and female subjects with type 1 and type 2 diabetes aged from 13 to 55 years (67). The DEPS-R scale is currently recognized as a valid screening tool for use in identifying individuals at-risk for developing ED in diabetic population (38, 67, 69–72). A total score ≥ 20 defined a cut-off at risk of ED in diabetic patients.

#### Module H of the Structured Clinical Interview for DSM-IV Axis I Disorder

Lifetime prevalence of ED according to DSM-5 criteria was assessed by means of the Module H modified, according to DSM-5 criteria, of the Structured Clinical Interview for DSM-IV Axis I Disorder (SCID-I, Research Version, Non-Patient Edition) (63, 73). It was possible to investigate the following diagnostic categories: Anorexia Nervosa, Bulimia Nervosa, Binge Eating Disorder, Unspecified Eating Disorder, and Other Specified Eating Disorder according to DSM-5 criteria (the modified version of Module H is available from authors).

#### Biochemical Evaluation

As for the biochemical evaluation, glycated hemoglobin (HbA_1_c) was assessed. The HbA_1_c assay provides an accurate, precise measure of chronic glycemic levels, and correlates with the risk of diabetes complications, thus representing the gold standard for monitoring glycemic control in patients with Diabetes Mellitus. According to the 2013 American Diabetes Association recommendations the assay was carried out on whole blood samples and HbA_1_c was measured using a G8 analyzer (THOSO Diagnostics, Tokyo, Japan) by high-performance liquid chromatography, aligned with International Federation of Clinical Chemistry standardization, according to Diabetes Control and Complications Trial/United Kingdom Prospective Diabetes guidelines. A value of HbA_1_c < 7% indicated a good glycometabolic control of the disease (74).

### Statistical Analysis

Characteristics of the sample were described using mean, median, and frequencies, as appropriate. Continuous variables distribution was studied observing normality plots, skewness, and kurtosis. Given the small sample size, only distributions passing the Kolmogorov–Smirnov test were analyzed with parametric tests. The outcome was the presence of depression, defined as a BDI-IA score ≥ 10, while our exposure of interest was the DEB severity indicated by the DEPS-R score which was the main predictor. Metabolic variables (HbA_1_c, continuous subcutaneous insulin therapy, treatment adherence, duration of diabetes, diabetes complications) were treated as covariates while gender, age, education, employment, thyroid disorder, BMI, and physical activity were treated as confounders. Differences between depressed and non-depressed individuals were assessed using Chi-square test, two-sample *t*-test, and Mann-Whitney *U* test as appropriate. Bivariate correlations between DEPS-R score and continuous variables (HbA_1_c, age, duration of diabetes, BMI) were tested using Spearman’s ρ, while Mann-Whitney *U* test was used when comparing DEPS-R score distribution between categories of dichotomous variables (gender, insulin therapy regimen, treatment adherence, diabetes complications). Crude and adjusted Odds Ratios (OR) and 95% confidence intervals were calculated using binary logistic regression. First, we performed univariate analysis to calculate crude ORs, then we built multivariate modeling. To study the chosen predictors in our sample as well as test our hypothesis, we chose to include in multivariate analysis every independent variable irrespectively of crude ORs, despite a less parsimonious final model. Three binary logistic regression models were performed; groups of variables were entered in succeeding steps according to our *a priori* hypothesis. In model A only confounders were added. In model B psychopathology variables (Lifetime DSM-5 ED diagnosis and DEPS-R score) were added. DEPS-R score was controlled for both confounders and Lifetime DSM-5 ED diagnosis. The latter was entered to partial out the effect of an established history of ED diagnosis as our hypothesis referred to disturbed eating as a continuous dimension which applies also at a subclinical level. Finally in model C metabolic covariates were added to study each metabolic predictor’s unique contribution to depression in the full rank solution and observe its impact on confounders and DEPS-R coefficients. When continuous variables were treated as independent variables we performed the Box-Tidwell Test to check that the assumption of linear relationship between predictor and logit of the outcome was not violated. The Hosmer–Lemeshow test was performed to check goodness of fit. Level of significance was set at < 0.05. The statistical analysis was conducted using IBM SPSS^®^ Statistics version 28.0.0.0.

## Results

### Characteristics of Participants

A total of 172 participants were included in the final sample (86 males and 86 females). Clinical and demographic characteristics are presented in Table 1. The mean age at recruitment was 36.76 years (SD = 9.87, range = 17–55). Most of the sample received higher education (*n* = 119, 69.2%) and was employed (*n* = 144, 83.7%). Medical and psychiatric comorbidities are listed in Table 2. Hashimoto’s thyroiditis was by far the most frequent comorbid disease (*n* = 31, 18.0%) followed by hypertension (*n* = 19, 11.0%) and dyslipidemia (*n* = 19, 11.0%). There were 8 participants receiving psychiatric care at recruitment: 6 for Depressive Disorders (3.5%) and 2 for Psychotic Disorders (1.2%). None of the participants was ever diagnosed or treated for eating disorders prior to study inclusion.

**TABLE 1:** Characteristics of participants.

	Total (*n* = 172) Mean ± SD or *n* (%)
Females	86 (50.0%)
Age, year	36.76 ± 9.87
Higher education	119 (69.2%)
Unemployed	28 (16.3%)
BMI, Kg/m^2^	23.42 ± 5.24*
Obesity**	9 (5.2%)
Physical activity in the last 3 months	126 (73.3%)
Thyroid disease	33 (19.2%)
Continuous subcutaneous insulin therapy	36 (20.9%)
Poor or less than sufficient treatment adherence	71 (41.3%)
Duration of diabetes, years	17.69 ± 10.82
HbA_1_c, %	7.70 ± 1.60*
Neurological, ocular, or renal complications	37 (21.5%)
Diagnosis of DSM-5 eating disorder	36 (20.9%)
DEPS-R score	10 ± 12*
DEPS-R ≥ 20	33 (19.2%)
BDI-IA score ≥ 10	61 (35.5%)

*Median ± IQR. **Defined as BMI ≥ 30 Kg/m^2^.

**TABLE 2:** Participants with comorbid diseases (excluding diabetes-related complications).

Diseases	*n* (%)
**Endocrine**	33 (19.2%)
Hashimoto’s thyroiditis	31 (18.0%)
Other endocrine disease	4 (2.3%)
**Cardiovascular**	**31 (18.0%)**
Hypertension	19 (11.0%)
Dyslipidemia	19 (11.0%)
Other cardiovascular disease	2 (1.2%)
**Gastrointestinal**	**13 (7.6%)**
Coeliac disease	5 (2.9%)
Gastroesophageal reflux disease	5 (2.9%)
Other gastrointestinal disease	3 (1.7%)
**Psychiatric**	**8 (4.7%)**
Depressive disorder	6 (3.5%)
Psychotic disorder	2 (1.2%)
Pulmonary*	**5 (2.9%)**
**Neurological**	**5 (2.9%)**
**Dermatologic**	**4 (2.3%)**
**Hematologic**	**3 (1.7%)**
**Gynecological**	**3 (1.7%)**

*Asthma was the only pulmonary disease on our sample. Groups of diseases by system are bolded.

#### Metabolic Characteristics

Overall glycemic control in the sample was poor with 120 participants (69.8%) having HbA_1_C superior to 7.0% at the last assessment. The median value of HbA_1_C in the whole sample was 7.7% (IQR = 1.6, range = 4.9–12.6). Moreover 37 (21.5%) participants had Diabetes Complications and 71 (41.3%) had a treatment adherence which was judged as either “poor” or “less than sufficient” by the treating physician. Mean duration of disease was 17.69 years (SD = 10.82, range = 0–43). There were 36 participants on CSIT (20.9%). The median BMI of the sample was 23.4 Kg/m^2^ (IQR = 5.2, range = 16.4–53.1), with 9 participants (5.2%) above the obesity threshold of 30 Kg/m^2^. Most of the sample was physically active in the 3 months prior to the assessment (*n* = 126, 73.3%).

#### Psychopathological Characteristics

**FIGURE 1:**
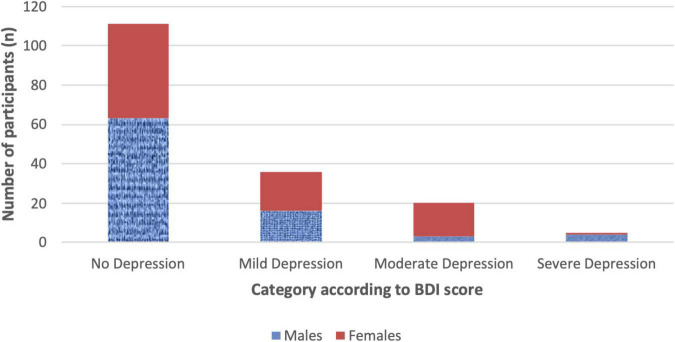
BDI scoring by gender.

There were 111 participants (64.5%) scoring 0–9 at BDI-IA indicating no/minimal depression, while scores of mild, moderate and severe depression were reported in respectively 36 (20.9%), 20 (11.6%), and 5 (2.9%) participants (Figure 1). The proportion of females with BDI-IA score indicating no, mild, and moderate-severe depression was, respectively, 55.8, 23.3, and 20.9%, compared to 73.3, 18.6, and 8.1% in males (χ^2^ = 7.311, p = 0.026).

A lifetime history of DSM-5 ED was detected in 20.9% of the sample (*n* = 36, Table 3); in particular in 34.9% of females compared to 7.0% of males (χ^2^ = 18.584, *p* < 0.001). Frequencies of ED type were as follows: 2 participants (1.2%) were diagnosed with Anorexia Nervosa (both females), 6 (3.5%) with Bulimia Nervosa (5 females, 1 male), 13 (7.6%) with Binge Eating Disorder (9 females, 4 males), 8 (4.7%) with Other Specified Feeding and Eating Disorder (5 females, 3 males) and *n* = 15 (8.7%) with Unspecified Feeding or Eating Disorder (15 females, no males). There were 8 participants (4.7%) who had a lifetime history of two different DSM-5 ED.

**TABLE 3:** Eating disorders diagnosed according to DSM-5 criteria in the study sample.

Disorder	*n* (%)
Anorexia nervosa	2 (1.2%)
Bulimia nervosa	6 (3.5%)
Binge eating disorder	13 (7.6%)
Other specified feeding and eating disorder	8 (4.7%)
Unspecified feeding or eating disorder	15 (8.7%)

There were 33 participants (19.2%) with a DEPS-R score ≥ 20. According to items scoring (Table 4) 56.4% of participants (*n* = 97) had a history of not taking enough insulin when overeating and 6.4% (*n* = 11) of skipping insulin dose after overeating. There were 25 participants (14.5%) who responded affirmatively about feeling fat when taking the whole prescribed insulin dose. Less common behaviors were self-induced vomiting (*n* = 6; 3.5%) and deliberate eating until ketonuria (*n* = 5; 2.9%), although 13 participants (7.6%) had a history of voluntary hyperglycemia to lose weight. The majority of the sample felt it was difficult to lose weight and control diabetes at the same time (*n* = 106; 61.6%) and 34 participants (19.8%) scored positively about preferring to lose weight rather than controlling diabetes. There was a weak significant positive correlation between DEPS-R and both HbA_1_C (ρ = 0.293; *p* < 0.001) and BMI (ρ = 0.337; *p* < 0.001), while correlations with age and duration of diabetes were non-significant (Table 5). Mann-Whitney *U* test (Table 6) revealed significantly higher scores of DEPS-R in females compared to males (*U* = 4700, *p* = 0.002); in patients with poor treatment adherence compared to patients with good treatment adherence (*U* = 4894.5, *p* < 0.001) and in those with diabetes complications compared to those without (*U* = 3188.5, *p* = 0.010). Contrary to our hypothesis, no significant difference in DEPS-R score was detected between patients on MDI regimen and patients on CSIT (*U* = 2240.0; *p* = 0.433).

**TABLE 4:** DEPS-R items endorsed.

Question	*n* (%)
Losing weight is an important goal to me	124 (72.1%)
Other people tell me to take better care of my diabetes	117 (68.0%)
I skip meals and/or snacks	111 (64.5%)
I feel that it’s difficult to lose weight and control my diabetes at the same time	106 (61.6%)
When I overeat, I don’t take enough insulin to cover the food	97 (56.4%)
Other people have told me that my eating is out of control	90 (52.3%)
I feel that my eating is out of control	87 (50.6%)
I alternate between eating very little and eating huge amounts	87 (50.6%)
I eat more when I am alone than when I am with others	77 (44.8%)
I avoid checking my blood sugar when I feel like it is out of range	55 (32.0%)
I would rather be thin than to have good control of my diabetes	34 (19.8%)
I feel fat when I take all of my insulin	25 (14.5%)
I try to keep my blood sugar high so that I will lose weight	13 (7.6%)
After I overeat, I skip my next insulin dose	11 (6.4%)
I make myself vomit	6 (3.5%)
I try to eat to the point of spilling ketones in my urine	5 (2.9%)

**TABLE 5:** Correlation between DEPS-R score and age, duration of diabetes, HbA_1_C, and BMI.

Continuous variable	*Spearman’s*ρ	*p*
Age at recruitment	−0.018	0.123
Duration of diabetes	0.029	0.710
HbA_1_C	**0.293**	**<0.001**
BMI	**0.337**	**<0.001**

*Significant values are bolded.*

**TABLE 6:** Distribution of DEPS-R score in categories of dichotomous variables.

Dichotomous variable	*DEPS-R Median (IQR)*	*Mann-Whitney U*	*p*
Categories (n)			
**Gender**		**4700.0**	**0.002**
Female (*n* = 86)	14 (13)		
Male (*n* = 86)	9 (9)		
**Insulin therapy regimen**		2240.0	0.433
MDI (*n* = 136)	11 (13)		
CSIT (*n* = 36)	11 (12)		
**Treatment adherence**		**4894.5**	**<0.001**
Poor or less than sufficient (*n* = 71)	15 (11)		
Sufficient, good, or excellent (*n* = 101)	9 (10)		
**Neurological, ocular, or renal complications**		**3188.5**	**0.010**
Presence of complications (*n* = 37)	14 (11)		
Absence of complications (*n* = 135)	10 (12)		

*n, number of subjects in each category. Categories and significant values are bolded.*

### Characteristics Associated With Depression

Differences between depressed and non-depressed individuals and crude ORs are presented in Table 7. Lifetime DSM-5 ED were significantly more prevalent in participants with depression compared to those without (34.4% vs. 13.5%; χ^2^ = 9.178, *p* = 0.002). The former scored also significantly higher on DEPS-R (17 ± 13 vs. 9 ± 9; *U* = 4.8290, *p* < 0.001). Rates of current DEB defined as DEPS-R ≥ 20 were significantly higher in depressed patients (36.1% vs. 9.9%; χ^2^ = 15.723, *p* < 0.001). No significant difference was detected regarding HbA_1_C levels (7.6 ± 1.8 vs. 7.8 ± 1.4, *U* = 3463.5, *p* = 0.651), duration of diabetes (18.3 ± 9.9 vs. 17.4 ± 11.3, *t* = −0.546, *p* = 0.586), poor treatment adherence (42.6% vs. 40.5%; χ^2^ = 0.011, *p* = 0.918) and diabetes complications (24.6% vs. 19.8; χ^2^ = 0.286, *p* = 0.593). In univariate analysis variables significantly associated with depression were female gender (OR: 2.17; 95% CI: 1.14–4.11; *p* = 0.026), lower education (OR: 2.92; 95% CI: 1.49–5.71; *p* = 0.003), Lifetime DSM-5 ED diagnosis (OR: 3.36; 95% CI: 1.57–7.17; *p* = 0.002) and DEPS-R score (OR: 1.10; 95% CI: 1.06–1.15; *p* < 0.001). None of the metabolic covariates were significantly associated with the outcome (Table 7).

**TABLE 7:** Association between predictors and depression in univariate analysis.

	BDI-IA ≤ 9 (*n* = 111)	BDI-IA ≥ 10 (*N* = 61)	Student’s t/Mann-Whitney U/Paerson’s χ ^2^	OR (95% CI)^a^
	
	Mean ± SD or *n* (%)	Mean ± SD or *n* (%)		
Age at recruitment, years	37.3 ± 10.2	35.7 ± 9.3	*t* = 1.041	0.98 (0.95–1.02)
**Female gender**	**48 (43.2%)**	**38 (63.2%)**	χ ^2^ = 4.979*	**2.17 (1.14**–4.11)*
**Lower education**	**25 (22.5%)**	**28 (45.9%)**	χ ^2^ = 9.026*	**2.92 (1.49**–5.72)*
Unemployed	15 (13.9%)	13 (21.3%)	χ^2^ = 1.063	1.68 (0.74–3.81)
**DSM-5 eating disorder**	**15 (13.9%)**	**21 (34.4%)**	χ ^2^ = 9.178*	**3.36 (1.57**–7.17)*
**DEPS-R ≥ 20**	**11 (9.9%)**	**22 (36.1%)**	χ ^2^ = 15.723**	**5.13 (2.28**–11.56)**
**DEPS-R score**	**9 ± 9** *^b^*	**17 ± 13** *^b^*	*U* = 4829.0**	**1.10 (1.06**–1.15)**
HbA_1_c, %	7.8 ± 1.4*^b^*	7.6 ± 18*^b^*	*U* = 3463.5	1.10 (0.85–1.42)
Duration of diabetes, years	17.4 ± 11.3	18.3 ± 9.9	*t* = −0.546	1.01 (0.98–1.04)
CSIT	22 (19.8%)	14 (23.0%)	χ^2^ = 0.082	1.21 (0.57–2.57)
Poor or less than sufficient treatment adherence	45 (40.5%)	26 (42.6%)	χ^2^ = 0.011	1.09 (0.58–2.05)
Neurological, ocular, or renal complications	22 (19.8%)	15 (24.6%)	χ^2^ = 0.286	1.32 (0.63–2.78)
BMI, Kg/m^2^	23.4 ± 5.0*^b^*	23.4 ± 5.5*^b^*	*U* = 3294.5	1.03 (0.96–1.10)
No physical activity	21 (19.4%)	18 (31.6%)	χ^2^ = 2.408	1.91 (0.92–3.98)
Thyroid disorder	20 (18.0%)	13 (21.3%)	χ^2^ = 0.104	1.23 (0.56–2.69)

*p < 0.05. **p < 0.001. ^a^Crude Odds Ratio and 95% Confidence Intervals (95% CI) calculated using binary logistic regression. ^b^Median ± IQR. Significant values and related variables are bolded.

### Multivariate Analysis

Results from simple logistic regression modeling are summarized in Table 8. In model A both female gender and lower education were significantly associated with the outcome, yielding adjOR respectively of 3.02 (95% CI: 1.39–6.56, *p* = 0.005) and 3.08 (95% CI: 1.39–6.82, *p* = 0.002). In model B after adding psychopathology variables the effect of female gender on depression was attenuated to non-significant level (adjOR = 1.64; 95% CI: 0.68–3.97, *p* = 0.272). Independent variables significantly associated with the outcome were DEPS score (adjOR = 1.08; 95% CI: 1.03–1.14, *p* = 0.003), which resulted a significant predictor even if adjusted for a Lifetime DSM-5 ED diagnosis, and lower education (adjOR = 3.22; 95% CI: 1.41–7.35, *p* = 0.005). Hosmer-Lemeshow test indicated good fitting of the data in the model. Nagelkerke *R*^2^ increased from 15.5% of model A to 26.5% of model B. In model C metabolic covariates were added except for treatment adherence and diabetes complications, given the strong collinearity with HbA_1_c (*U* = 6508.0, *p* < 0.001) and duration of diabetes (*t* = −4.117, *p* < 0.001), respectively. As in univariate analysis, none of these variables yielded significant adjOR, with only a marginal increase in the Nagelkerke *R*^2^. We did not observe any alteration of neither the direction nor the significance of the coefficients compared to model B. In this final model, every point scored in DEPS-R was associated with a 1.09-fold increase of the odds of depression in T1DM adult patients. Also, patients with lower education were 3.60 times more likely to have depression than patients with higher education. The final Nagelkerke *R*^2^ was weak (28.4%).

**TABLE 8:** Multivariate logistic regression models for the association between the presence of depression (dichotomous dependent variable) and DEPS-R score (main exposure of interest, continuous).

	Model A			Model B			Model C		
Independent variable (c/reference)*^a^*	B	SE	OR (95% CI)	B	SE	OR (95% CI)	B	SE	OR (95% CI)
Age at recruitment (c)	−0.034	0.019	0.97 (0.93–1.00)	−0.019	0.020	0.98 (0.94–1.02)	−0.022	0.022	0.98 (0.94–1.02)
Female gender (Male Gender)	1.104	0.396	3.02 (1.39–6.56)*	0.495	0.451	1.64 (0.68–3.97)	0.525	0.467	1.69 (0.68–4.23)
Lower education (Higher Education)	1.124	0.406	3.08 (1.39–6.82)*	1.170	0.420	3.22 (1.41–7.35)*	1.280	0.437	3.60 (1.53–8.47)*
Unemployed (Employed or Student)	0.411	0.470	1.51 (0.60–3.79)	0.262	0.491	1.30 (0.50–3.40)	0.386	0.519	1.47 (0.53–4.07)
BMI (c)	0.014	0.041	1.01 (0.94–1.10)	−0.066	0.047	0.94 (0.85–1.03)	−0.065	0.048	0.94 (0.85–1.03)
No physical activity (Yes physical activity)	0.312	0.421	1.37 (0.60–3.12)	0.437	0.442	1.55 (0.65–3.68)	0.493	0.449	1.64 (0.68–3.95)
Presence of thyroid disease (Absence of Thyroid Disease)	−0.203	0.513	0.82 (0.30–2.23)	−0.047	0.542	0.95 (0.33–2.76)	−0.060	0.548	0.94 (0.32–2.76)
Presence of lifetime DSM-5 ED (Absence of Lifetime DSM-5 ED)	/	/	/	0.564	0.520	1.76 (0.63–4.87)	0.549	0.535	1.73 (0.61–4.94)
DEPS score (c)	/	/	/	0.080	0.027	1.08 (1.03–1.14)*	0.087	0.028	1.09 (1.03–1.15)*
HbA_1_c (c)	/	/	/	/	/	/	−0.254	0.178	0.78 (0.55–1.10)
Duration of diabetes (c)	/	/	/	/	/	/	0.006	0.021	1.01 (0.97–1.05)
CSIT (MDI regimen)	/	/	/	/	/	/	0.395	0.481	1.49 (0.58–3.81)

Data are presented as OR with 95% Confidence Intervals (95% CI), regression coefficient (B), and Standard Error (SE). Model A: confounders (age, gender, education, employment, BMI, physical activity, thyroid disorders). Model B: model A + psychopathology variables (Lifetime DSM-5 ED diagnosis and DEPS-R score). Model C: model B + metabolic covariates (HbA_1_C, duration of diabetes, insulin treatment regimen). ^a^For dichotomous categorical independent variables the reference group is presented, while continuous independent variables are marked with the letter c. *p < 0.05. Significant values are bolded.

## Discussion

The present study was aimed at investigating a sample of 172 outpatients affected by T1DM (age: 17–55 years; mean duration of illness: 17.69 years) attending a diabetology clinic to assess the following: the current prevalence and severity of depression and DEB, lifetime prevalence of ED according to DSM-5 criteria, association between DEB and depression and impact of DEB and depression on diabetes control.

Overall, most of the sample studied was characterized by poor glycemic control: indeed, at the last assessment, 69.8% of participants displayed HbA_1_c levels (parameter measuring blood sugar levels over the previous 2–3 months) exceeding 7%, indicative of poor glycemic control. The presence of diabetic complications (neurological, ocular, or renal) was reported in 21.5% of cases, whilst poor or inadequate treatment compliance was reported, based on clinical observations, in 41.3% of patients. High clinically relevant levels of depression were detected in 35.5% of the sample (26.7% of males and 44.2% of females). This finding is in line with those reported by other authors who highlighted how depression is a singularly common psychopathological dimension amongst patients with T1DM, implicating a 2.4 to 3.8-fold higher risk compared to a non-diabetic population (75). Likewise, the finding of higher rates of depression amongst females adheres perfectly to the data in literature reporting overall a 2-fold increase in risk of depression in the female gender compared to males, both in the general population (1) and in the population of diabetic patients (14, 15). In the most recent meta-analysis Farooqi et al. (76) reported a prevalence of depression of 22% in T1DM patients which is below the prevalence in our sample. This could be explained by the fact that most of the studies to date were conducted on samples of adolescents and youth while depressive disorders tend to occur more often later in life (58). Moreover, our study was conducted in a specialist care setting which was associated with higher prevalence rates in the aforementioned meta-analysis (76). Finally, the use of self-report questionnaires to assess depression in patients with diabetes have been found to be associated with higher rates compared to standardized diagnostic interviews (15), possibly underlying an overestimation of the depressive dimension due to the difficulty of distinguishing between a true depressive state and a condition of distress linked to diabetes management. To this regard, Fisher et al. raised the issue of the complex interpretation of data deriving from self-administered questionnaires to assess for depression, comparing the data deriving from these questionnaires with findings obtained by means of the Mood Disorders Module of Structured Clinical Interview, considered the diagnostic gold standard, thus identifying a high rate of false positives (20). It should however be considered that issues relating to reliability and validity were also raised about the use of structured interviews (77). Wisting et al. (40) who conducted a study on a comparable sample of T1DM adults, found a much lower prevalence of depression (6.2%) using the Hospital Anxiety and Depression Scale (HADS) (78). Even considering only moderate-to-severe cases, therefore using a cut-off of 19 in the BDI-IA [which is more conservative than the cut-off of 16 identified by Lustman et al. (79) in their validation study on diabetic outpatients], the prevalence of depression in our sample is still more than twice (14.5%) the prevalence found by Wisting et al. We think this finding is of interest, especially because both studies were conducted in a specialist care setting and both samples showed comparable rates of DEB. Part of this discrepancy is probably due to the different self-report questionnaire used. HADS was consistently more specific and less sensitive compared to BDI-IA in studies on patients with Human Immunodeficiency Virus (80) and Chronic Obstructive Pulmonary Disease (81). Although Wisting et al., did not report socio-demographic data, we can also hypothesize that different socio-demographic characteristics might have driven the higher prevalence of depression in our sample (i.e., less education).

According to DSM-5 criteria, the overall lifetime prevalence of ED was 20.9%, 7% in males and 34.9% in females. A total of 19.2% of the sample, 10.5% of males and 27.9% of females, achieved or exceeded a score of ≥20 at DEPS-R, a cut-off used to identify a risk of ED amongst diabetic patients. On examining prevalence data of DEB assessed by means of DEPS-R in greater detail, 56.4% of the sample had a history of “not taking enough insulin when overeating”; 6.4% (*n* = 11) of “skipping insulin dose after overeating”; 14.5% replied in the affirmative to “feeling fat when taking the whole prescribed insulin dose.” These findings are in line with literature data relating to the prevalence of ED and DEB in diabetic patients vs. the general population (38, 42, 45, 46, 49). Wisting et al. (40) found a comparable prevalence of DEB in their T1DM adult outpatients’ sample (20.3%), using the same assessment instrument. Compared to males, the female members in our sample displayed a significantly higher prevalence of lifetime ED according to DSM-5 criteria and higher scores at DEPS-R, indicative of an increased severity of DEB. Data present in literature support the finding of a generalized higher prevalence and an increased risk of ED in female adolescents and adults, although a relatively high prevalence is also observed amongst males (82, 83). Indeed, the finding in our study of higher scores in the female gender at DEPS-R is in line with data reported in the context of validation studies conducted on the rating scale (67, 69, 71, 72).

In the present study DEB was also significantly associated with variables related to poor metabolic control: (i) we found a positive signification correlation with HbA_1_C (although with a weak effect size); (ii) we found higher DEPS-R scores in patients with poor or less than sufficient treatment adherence compared to those with sufficient, good or excellent treatment adherence; (iii) we found higher DEPS-R scores in subjects with diabetes complications compared to those without. Eisenberg Colman et al., reported an association between DEB and higher HbA_1_C and worse treatment adherence in a sample of adolescents with T1DM (84). Our study replicates this finding in a sample of adult outpatients, in line with the study of Wisting et al. (40). Contrary to our hypothesis, no difference in DEB was found when comparing patients on CSIT with patients on MDI regimen. Prior research has consistently shown an improvement in psychological wellbeing with CSIT (85). In a recent study, adolescents with T1DM on CSIT scored significantly lower on the Binge Eating Scale (BES) compared to those on basal-bolus regimen (86). In our sample, using an instrument specifically constructed to assess DEB in T1DM patients, this association was not replicated. We can hypothesize that BES (87), a scale designed to assess binge eating in obese patients, has only limited validity to assess DEB in T1DM patients as most of insulin-related disordered behavior is not included. Finally, BMI was significantly positively correlated with DEPS score. In other studies, higher BMI indexes were found to underlie an increased drive to lose weight, resulting in dieting, negative affect, and disordered eating (38, 39, 88). BMI should therefore be deemed a potential risk factor for disordered eating in patients affected by T1DM (88).

As hypothesized prevalence rates of both lifetime DSM-5 ED and current DEB were significantly higher in depressed patients compared to non-depressed (respectively 34.4% vs. 13.9% and 36.1% vs. 9.9%). Of note no patient was ever diagnosed or treated for ED prior to study inclusion. This finding supports the importance of screening for ED and DEB in T1DM patients with depressive symptoms, as other authors reported a worse course of the disease and poor response to treatment of the depressive disorder in case of comorbidity (54). Future research should focus on the longitudinal course and therapeutic targeted interventions for depression and ED comorbidity in T1DM patients.

Contrary to our hypothesis, we did not detect any significant association between the presence of depression and variables related to poor glycemic control. Specifically, no association was revealed with HbA_1_c levels. There are conflicting data in literature regarding the link between indicators of glycemic compensation and depression, with some studies that highlight a poorer compensation in depressed subjects (19) and others that do not (40, 57). It is worthy of note that in comparison with other studies in which indicators of metabolic compensation and levels of depression were evaluated contextually (19), in the present study HbA_1_c levels related to the last control undertaken over the previous 3 months, whilst the reference time frame for assessment of depression using the BDI-IA scale was the previous 2 weeks. The findings obtained in our study agree with those reported by Fisher et al. who raised the issue of the methodological limitation of a non-contextual evaluation to justify the lack of association between depression and Hb1Ac levels (20).

Multivariate analysis revealed higher scores at DEPS-R increased the odds of depression at BDI independently of metabolic covariates (HbA_1_c, CSIT, duration of diabetes) and confounders (gender, age, education, employment, thyroid disorder, BMI, and physical activity). In particular, there was an increase in the odds of depression by 9% for every increase of DEPS-R by each one point. Lower education was also independently associated with the outcome. This finding highlights the independent association between depression and DEB in patients with T1DM, bearing in mind, however, the cross-sectional design of the study. Several authors had previously reported a link between levels of depression and DEB (38, 39). In the context of a study conducted on a sample of female adolescents with T1DM, Colton et al. highlighted how subjects manifesting a more significant depressive symptomatology also displayed a higher incidence of DEB (39). Wisting et al., found a medium correlation between DEPS-R and HADS Depression in their T1DM adults sample (40). In our study, we extend these findings by showing with multivariate analysis an independent association between DEB and depression in our sample. Interestingly, Bächle et al. (89) conducted a study on T1DM patients aged 18–21 years old. In their sample, a positive SCOFF questionnaire (a screening questionnaire for ED) increased depression severity independently of several confounders (age, diabetes duration, BMI, socioeconomic status, family structure). Our results strengthen their findings, given that in our study: (i) DEB was assessed with a psychodiagnostics questionnaire validated on diabetic outpatients; (ii) we included different covariates in multivariate analysis, in particular those related to glycemic control; (iii) we recruited adult subjects with a mean age of 36.76 years old. Finally, the finding observed in the present study of an association between lower education and levels of depression is in line with data reported in literature, thus confirming this association (90).

The present study should, however, be interpreted considering a series of methodological limitations. Firstly, the cross-sectional design of the study did not allow the causal relationship, and therefore the direction of relationship between the factors investigated, to be determined. Secondly, the limitation of assessing depression using a self-assessment tool which, as highlighted previously, particularly in populations affected by a chronic medical condition such as diabetes, may not be capable of discriminating between a depressive state and a condition of distress correlated with the underlying illness. Thirdly, the non-contextual assessment of the depressive dimension and indicators of metabolic compensation. Fourthly, the absence of a control group. Lastly, the variables included in the final model explained only 28.4% of the variance in depression in our sample. Future research should include other variables (i.e., family history of mood disorders, personality traits, life events, childhood trauma) to find other significant predictors of depression in T1DM adults outpatients. Despite the aforementioned limitations, to our knowledge, this is the first study to examine the independent association between depression and DEB in T1DM adult outpatients, taking into account a numerous set of covariates and using a validated psychodiagnostic tool tailored to the diabetic population to assess DEB.

## Conclusion

This study highlights a statistically significant and independent association between DEB and depression in a population of adult patients affected by T1DM. Prevalence of lifetime ED and current DEB and depression were elevated in our sample. Bearing in mind the diagnostic, clinical, and therapeutic implications of comorbidity between diabetes and depression and diabetes and ED, and the singularity of the forms of presentation of depressive and ED in diabetic subjects, routine analysis of the depressive dimension and ED using purpose-developed screening tools of proven validity should be deemed essential in this patient population. Furthermore, the complex management of comorbidity between diabetes, depression and ED implies a need for timely and efficient structured multidisciplinary intervention programs aimed at managing the physical and mental health of patients and minimizing the risk of short- and long-term complications linked to the comorbid conditions. The definition of specific therapeutic interventions for management of DEB could have positive effects on the control of diabetes and on the levels of depression in this population of patients. In the light of the previously acknowledged methodological limitations, the significance of the findings reported in this study suggest the need for further studies to be carried out on a larger patient population using a longitudinal study design and accurate method of evaluation aimed at yielding a wider and more reliable investigation of the complex relationship between diabetes, depression, ED, and DEB.

## Data Availability Statement

The raw data supporting the conclusions of this article will be made available by the authors, without undue reservation.

## Ethics Statement

Ethical review and approval was not required for this study on human participants in accordance with the local legislation and institutional requirements. Written informed consent to participate in this study was provided by the participants’ legal guardian/next of kin.

## Author Contributions

FP planned the study and drafted and revised the manuscript. FS drafted the result section and contributed to analyzing and interpreting the findings. VD and ED contributed to the planning of the study and collected data. FF, MG, RC, MT, EC, and EL collected somatic data. EN and PG contributed to analyzing and interpreting the findings. AL, LL, MM, PP, GS, and AC reviewed the manuscript and contributed to the discussion. BC and FV contributed to the planning of the study, reviewed the manuscript, and contributed to the discussion. All authors read and approved the final manuscript.

## Conflict of Interest

The authors declare that the research was conducted in the absence of any commercial or financial relationships that could be construed as a potential conflict of interest.

## Publisher’s Note

All claims expressed in this article are solely those of the authors and do not necessarily represent those of their affiliated organizations, or those of the publisher, the editors and the reviewers. Any product that may be evaluated in this article, or claim that may be made by its manufacturer, is not guaranteed or endorsed by the publisher.

## Abbreviations

BDI-IA: beck depression inventory–IA version
BES: binge eating scale
BMI: body mass index
CSIT: continuous subcutaneous insulin therapy
DEB: disturbed eating behaviors
DEPS-R: diabetes eating problems survey—revised
DSM-5: diagnostic and statistical manual of mental disorders5TH edition
ED: eating disorders
HADS: hospital anxiety and depression scale
HbA_1_c: glycated hemoglobin A1c
IQR: interquartile range
MDI: multiple daily injections
OR: odds ratio
SCID-I: structured clinical interview for DSM-IV axis I disorder
SD: standard deviation
T1DM: type 1 diabetes mellitus
T2DM: type 2 diabetes mellitus.
